# The Reliability of the DEM Test in the Clinical Environment

**DOI:** 10.3389/fpsyg.2018.01279

**Published:** 2018-07-25

**Authors:** Alessio Facchin, Silvio Maffioletti

**Affiliations:** ^1^Department of Psychology, University of Milano-Bicocca, Milan, Italy; ^2^COMiB – Research Center in Optics and Optometry, University of Milano-Bicocca, Milan, Italy; ^3^NeuroMi - Milan Center for Neuroscience, Milan, Italy; ^4^IRSOO – Institute for Research and Studies in Optics and Optometry, Vinci, Italy; ^5^Optics and Optometry, University of Turin, Turin, Italy

**Keywords:** DEM test, reliability, test–retest, learning effect, psychometrics, clinical assessment

## Abstract

The developmental eye movement (DEM) test is a practical and simple method for assessing and quantifying ocular motor skills in children. Different studies have previously assessed the reliability of the DEM test and they have generally found high values for vertical and horizontal time, whereas those for Ratio and Errors were medium and low, respectively. In the second application of test were found an improvement in performance in all subtests. Our aim was to evaluate the reliability of the DEM test using seconds and percentile scoring and looking in depth at the improvement in performance when the test is repeated. We tested the reliability of the DEM test on a group of 115 children from the 2nd to the 5th grade using different statistical methods: correlations, ANOVA, limits of agreement for results expressed in seconds and as percentile scoring and pass-fail diagnostic classification. We found high reliability with excellent values for vertical and adjusted horizontal time, medium-to-high for ratio and medium for errors. We have re-confirmed the presence of a significant improvement of performance on the second session for vertical time, horizontal time and ratio. The stability of binary classification of Pass–Fail criteria appears to be medium. We found high reliability for the DEM test when compared with the published results of other research but the improvement of performance, the learning effect was still present, but at a lower level than previously found. With the awareness of these limitations the DEM test can be used in clinical practice in evaluating performance over time.

## Introduction

The developmental eye movement (DEM) test is a practical and simple method for assessing and quantifying ocular motor skills in children. The DEM test allows clinicians interested in vision to obtain an easy quantitative measurement of ocular-movement skills by means of a psychometric test. The task consists of naming numbers in a simulated reading-like condition (Garzia et al., 1990).

The DEM test comprises three different individual plates. Two plates contain regularly spaced numbers, each displaced in two different columns for vertical reading (Card A and Card B). These determine the automaticity of number-naming ability. The third plate contains unevenly spaced numbers, displaced in sixteen different lines for horizontal reading (Card C). This evaluates number naming in a reading-like task. The ratio score is calculated by dividing the adjusted horizontal time, corrected for errors, by the vertical time. The vertical time, adjusted horizontal time, ratio, and error scores are compared with the published normative dataset and used to identify dysfunctions related to either number naming, ocular motor skills, or a combination of the two.

The choice of a psychometric test such as DEM is determined by considering the three factors that characterize its properties: validity, reliability, and normative values (Anastasi and Urbina, 1997; Facchin et al., 2011). The validity of the DEM test in assessing ocular movement has been the subject of some discussion (Medland et al., 2010; Webber et al., 2011). Some studies concluded that DEM did not measure ocular movements (Ayton et al., 2009). Conversely, others studies have evaluated the validity of the DEM test (Garzia et al., 1990; Facchin et al., 2011) and, although it did not seem to correlate directly with pure eye movement parameters, it was related with different aspects of reading performance and it is useful in clinical practice (Powers et al., 2008; Ayton et al., 2009; Palomo-Álvarez and Puell, 2009). Even where there are influences from many cognitive processes, such as sustained attention (Coulter and Shallo-Hoffmann, 2000), number recognition and retrieval, visual verbal integration time, speaking time and visuo-spatial attention (Facchin et al., 2011), the DEM test provided the potential to measure visual skills related to ocular movements in a reading-like condition. In actual fact, normative values are available for the English (Richman and Garzia, 1987), Spanish (Fernandez-Velazquez and Fernandez-Fidalgo, 1995; Jimenez et al., 2003), Cantonese (Pang et al., 2010), Japanese (Okumura and Wakamiya, 2010), Portuguese (Baptista et al., 2011), Italian (Facchin et al., 2012), Mandarin (Xie et al., 2016), and Latvian (Serdjukova et al., 2016) languages.

Test–retest reliability means that a test should produce the same score for each subject when it is performed twice without apparent changes in the variable measured (Urbina, 2004; Kline, 2014). As applied to the DEM test, reliability was tested several times over periods of years. The test manuals (Richman and Garzia, 1987; Richman, 2009) reports that reliability was tested on forty subjects from grades one through seven and gives the following correlation coefficients (Pearson *r*): for vertical time, *r* = 0.89, *p* < 0.001; for adjusted horizontal time, *r* = 0.86, *p* < 0.01; for ratio, *r* = 0.57 *p* < 0.05; for errors, *r* = 0.07 n.s. Taken together, these data show that the DEM test has good reliability (test–retest correlation) for vertical and horizontal time, but medium for ratio, and low for errors. Santiago and Perez (1992) have replicated these results, finding only a higher value for errors.

Rouse et al. (2004) tested a group of 30 3rd grade children, and retested them 2 weeks later. They found that vertical and adjusted horizontal time both have fair to good repeatability, whereas that for the ratio score was found to be poor. It is necessary to take into account that a single classroom was used in the study and not a stratified sample. Interestingly, in this study, it was introduced the concept of limits of agreement with a corresponding graphical representation (Altman and Bland, 1983; Bland and Altman, 1986).

Tassinari and DeLand (2005) tested two groups, in office and in school environments. The correlation coefficients were higher than those previously found and, remarkably, good agreement was reported between test and retest in terms of pass-fail classification only for the office group.

Orlansky et al. (2011) performed a more extensive evaluation of reliability in a multi-center study. More than 180 subjects were tested in two sessions, in each of which they were each evaluated three times. The most important results are the fair to good correlation coefficients between-session for both the vertical and horizontal scores and the poor results for the ratio and error scores. Regarding pass-fail classification, the proportion of subjects who stayed in the same classification was in the range from 71 to 100% for both vertical and horizontal scores. For ratio and error scores, the proportion of subjects that remained classified as pass or fail was between 47 and 100%. However, they found that children in this age range could show improvements in all four test scores without any intervention. Finally, it was concluded that clinicians should be careful about using the DEM test for diagnosis or to monitor the effectiveness of treatment. The pass/fail analyses were performed based on two cut-offs at the 16th and 30th percentiles. The researchers administered three parallel versions of the DEM test (the same 80 numbers in different sequences) in order to eliminate implicit or explicit memorization of the numbers. In a clinical setting it is impossible to use parallel versions because the original test was not designed to have such forms. Indeed, from a theoretical point of view, parallel forms seem plausible and the normative data appear to be equally valid.

In the last case, the parallel form of test reliability was in fact evaluated, but it did not represent the true test–retest reliability of a single version of the clinical test. Moreover, unlike manual instructions, the vertical time for errors was also corrected; when the original manual (and the large part of norms) did not require this correction to be performed (the scoresheet in the 1987 manual reported this calculation incorrectly). Again, the multiple repetition of test within each session could affect the true between session test–retest reliability.

In the studies mentioned previously, the general term reliability has incorporated concepts and scores derived from the agreement term. The border between the concepts of reliability and agreement may not always be clear (Costa Santos et al., 2011a,b), and for this reason we discuss reliability and agreement separately.

Broadly speaking, from a pure psychometric point of view, the reliability is the correlation coefficient between test and retest (Anastasi and Urbina, 1997; Urbina, 2004). On the other hand, it provides information regarding the ability of the score to distinguish between subjects (Kottner and Streiner, 2011). The DEM test shows a high reliability, with the exception of ratio which shows a medium to high relationship. Correlation refers to the linear relationship with two sessions of administration, but it can provide nothing regarding the changes with respect to the absolute score. In fact, this concept was better explained by the agreement term, which represents the similarity of scores, and judgment or diagnoses with respect to the degree in which they differ (Kottner and Streiner, 2011). Rouse et al. (2004) and Orlansky et al. (2011) have shown that the true problem with the DEM test appears to be the improvement between sessions, which can be defined as a form of lack of agreement. This improvement was also defined as learning effect (Orlansky et al., 2011) and reported in terms of mean change and its respective limits of agreement (Altman and Bland, 1983).

Based on the aforementioned considerations, when compared with the study by Orlansky et al. (2011), using a single test, we predict an equal or higher reliability, but a low agreement expressed with a high learning effect (high bias and wider limits of agreements). Different comparisons were performed with all other reliability studies in order to assess and compare reliability and agreement.

Consequently, in performing the present study we have three aims. Firstly, we wanted to test the reliability, quantify the learning effect and assess the agreement between sessions using only one established classification criterion and only one version of the test as used in clinical practice. Secondly, from a clinical and rehabilitation point of view, because DEM scores have previously been observed to improve between sessions in absence of intervention, we wanted to calculate the minimum amount of change that needs to be observed to consider the change a real change using percentile score. Thirdly, considering the recent needs of replication studies (Open Science Collaboration, 2015), we wanted to replicate the results of previous studies on DEM reliability involving a different population and norms.

## Materials and Methods

### Subjects

Children were taken from a school screening program performed in the “V.Muzio” public school in Bergamo, north of Italy. Only children with written informed consent from their parents to take part in the study were enrolled (Facchin et al., 2011, 2012). All participants were selected on the basis of the following criteria: they were required to use their glasses or contact lenses (if required) during testing; to have a monocular visual acuity at distance of at least 0.63 decimal (20/32 with Goodlite n. 735000 table), to have a near binocular visual acuity of at least 0.8 decimal (20/25 with Goodlite n. 250800 table); and not to present binocular anomalies (strabismus) at cover test and distance and near phoria in a normal range (±4 at distance and ±6 at near) measured with a Thorington technique (Rainey et al., 1998; Scheiman and Wick, 2013). Testing was performed in two sessions. Subjects who performed in only one session were excluded. 135 children from two primary schools in the north of Italy were screened, but only 115 met the required inclusion criteria (three participants were excluded for strabismus, eight for lower monocular distance visual acuity, nine for the absence of second session test; see **Table 1** for details of the final participants). The study was carried out in accordance with the guidelines given in the Declaration of Helsinki and the school council of the “V.Muzio” school approved the procedure.

**Table 1:** Sample description subdivided by grade and gender (M, male; F, female).

Grade	Mean age	Gender	*n*
2nd	7 years and 6 months	M	16
		F	9
3rd	8 years and 5 months	M	14
		F	15
4th	9 years and 10 months	M	22
		F	10
5th	10 years and 7 months	M	12
		F	17

Total			115

### Tests and Procedures

A short description of tests and procedures is given below.

Four cards comprise the DEM test: the pretest card, two vertical cards (A and B) and one horizontal card (C). The test was administered using the methodology given in the DEM manual. The vertical time represents the sum of that spent on naming the number printed on the two cards, A and B. The vertical time returns the time required to read 80 numbers organized vertically. The adjusted horizontal time represents the time required for card C corrected for omission or addition errors. The adjusted horizontal time reflects the time required to read the 80 numbers organized in a horizontal pattern, together with that needed to perform saccadic movements. Dividing the adjusted horizontal by the vertical time, the ratio score was calculated. This is used to assess ocular motility dysfunction. The total number of errors returns the accuracy of reading of card C. Italian normative tables (Facchin et al., 2012) were used to determine the percentile score for vertical time, adjusted horizontal time, ratio and error.

The DEM test was administered as reported in the manual on an inclined reading desk set at 40 cm, with constant illumination and without noise. The tests were administered in two different sessions, separated by between 14 and 20 days, in the same room, for every subject who completed the test in the first session.

### Statistical Methods

We have analyzed all aspects of test–retest reliability and agreement between the two measurements as a function of time. Wherever possible, our data were compared with the results obtained in other published research. In order to look at the results from a meaningful clinical viewpoint, additional analyses were applied using percentile scoring.

Firstly, because previous studies used three different correlation indexes (Richman and Garzia, 1987; Rouse et al., 2004; Tassinari and DeLand, 2005; Richman, 2009; Facchin et al., 2011; Orlansky et al., 2011) in order to perform inter-study comparison, the test–retest reliability for DEM was analyzed using: Pearson r correlation, partial correlation (adjusted for age) and intra class correlation (ICC) using the average score and One-Way model (McGraw and Wong, 1996). Confidence intervals for correlations were calculated following a specific procedure (Zou, 2007; Diedenhofen and Musch, 2015), and ICC and Cohen’s *K* difference were also calculated and reported using a specific methodology (Dormer and Zou, 2002; Ramasundarahettige et al., 2009).

Because Orlansky et al. (2011) have performed the test–retest evaluation with three repetitions in each session (30–90″ distance) in two sessions (1–4 weeks apart), from this study, only the first administration of each session was taken into account for comparison of correlation coefficients. According to the study of Fleiss and Cohen (1973) and the study of Viera and Garrett (2005), interpretation of correlation coefficients, Kappa and AC_1_ was based on five steps each of 0.2 points between 0 and 1 with the respective subdivision: low, low to moderate, moderate, moderate to high and high.

Secondly, in order to test the agreement, we calculated and plotted the Bland – Altman 95% limits of agreement (LoA; 1.96 ^∗^ SD) that gives the value and the range of differences between the test and re-test scores (Bland and Altman, 1986). If the test is truly reliable, differences outside of LoA limits have only 5% of occurrence. These limits have an error margin and consequently their respective confidence intervals (95% CI) were calculated. With these data expressed in seconds and in percentiles we can estimate the minimum change necessary in the second session to have a statistical confirmation of amelioration over two sessions of administration is due to a treatment and not to lack of agreement. In order to evaluate the mean bias between sessions, a repeated measure ANOVA was applied to each specific subtest.

To quantify the magnitude of the improvement over time, we proposed a simple index of learning effect, adapted to reliability. This index was calculated for each DEM subtest and can be summarized as:

$$\text{Learning Effect}(\%)=100^{*}\frac{\text{ReTest Mean}–\text{Test Mean}}{\text{Test Mean}}$$

where,

*ReTest Mean* = the mean value of all subjects in the second session,

*Test Mean* = the mean value for all subjects in the first.

The learning effect can give us an absolute mean percentage of improvement (in seconds). For clinical use, it is better to know the same effect scored in percentile in order to determine whether there is a significant amelioration over time. Finally, a standard error of measurement expressed as the standard deviation of errors of measurement that are associated with test reliability was calculated using the formula (Rouse et al., 2004):

$${Se}_{m}=SD\sqrt{1-r_{xx,}}$$

where,

Se_m_ = standard error of measurement,

SD = standard deviation,

*R*_xx_ = reliability of the test.

Thirdly, in order to evaluate and compare the agreement between sessions of the DEM test classification using pass–fail cut-off criteria, the Cohen’s Kappa (Fleiss et al., 1969) and the AC_1_ index (Gwet, 2008) were applied. Kappa was selected for the comparison of studies and AC_1_ was applied in order to avoid the paradoxical results found using Kappa index (Gwet, 2008). Before calculating Kappa and AC1, for each subject, a percentile scoring through DEM test specific Italian norms were calculated. In previous studies and in the manual, two criteria were used. The first refers to the first edition of manual (version 1/1987, 30th percentile criterion), whilst the second refers to the new edition (version 2; 2009, 16th percentile criterion). In order to be aligned with other Italian national psychoeducational criteria used in the cognitive evaluation of children, we applied the cutoff at the 16th percentile (Associazione Italiana Dislessia, 2007). If vertical time, adjusted horizontal time, ratio and errors presented a score that was equal or below the 16th percentile, it was marked as “fail.” If the score was above the 16th percentile, it was marked as “pass.” Data were analyzed using R statistical environment and specific packages (R Core Team, 2017).

## Results

### Reliability

The different correlation coefficients for test–retest reliability were determined and these are listed in **Table 2**. The results show high values for vertical time and adjusted horizontal time, and moderate to high for ratio and errors. This pattern was confirmed by partial correlation when the component due to age was removed. The ICC correlations also confirmed the good repeatability of all variables. Moreover, the confidence intervals are very small and the values vary from medium-high to high.

**Table 2:** Correlations, partial correlations and intra-class correlation coefficients (ICC) with relative 95% confidence intervals for the four DEM subtests.

	Pearson correlations		Partial correlations		Intraclass correlations	
			
	*r* (95% CI)	*p*	*r*_p_ (95% CI)	*p*	ICC (95% CI)	*p*
VT	0.933 (0.905 to 0.953)	<0.0001	0.902 (0.861 to 0.930)	<0.0001	0.932 (0.904 to 0.953)	<0.0001
AHT	0.901 (0.860 to 0.931)	<0.0001	0.816 (0.742 to 0.873)	<0.0001	0.892 (0.848 to 0.924)	<0.0001
Ratio	0.668 (0.552 to 0.758)	<0.0001	0.597 (0.459 to 0.701)	<0.0001	0.649 (0.529 to 0.743)	<0.0001
Errors	0.692 (0.582 to 0.776)	<0.0001	0.643 (0.524 to 0.742)	<0.0001	0.692 (0.583 to 0.776)	<0.0001


**VT, vertical time; AHT, adjusted horizontal time.**

The different studies on the repeatability of the DEM test used different correlation coefficients. To enable comparison, in the case of the studies of Richman and Garzia (1987) and Rouse et al. (2004), the evaluation was performed with the Pearson correlation coefficient, and for the study of Tassinari and DeLand (2005) and Orlansky et al. (2011) using the ICC (**Tables 3**, **4**)

**Table 3:** Pearson correlation coefficients comparison between this study and those of Rouse et al. (2004) and Richman (2009).

	This work	DEM manual	Difference (95% CI)	*p*	Rouse et al. (2004)	Difference (95% CI)	*p*
Subjects (*n*)	115	40			30		
VT	0.93	0.89	0.04 (-0.019 to 0.132)	n.s.	0.60	0.33 (0.138 to 0.625)	<0.001
AHT	0.90	0.86	0.04 (0.036 to 0.155)	n.s.	0.55	0.35 (0.136 to 0.665)	<0.0001
Ratio	0.66	0.57	0.09 (-0.124 to 0.362)	n.s.	0.27	0.39 (0.064 to 0.772)	<0.05
Errors	0.69	0.07	0.62 (0.297 to 0.948)	<0.01			


**VT, vertical time; AHT, adjusted horizontal time.**

**Table 4:** ICC correlation coefficients comparison between this study and those of Tassinari and DeLand (2005) and Orlansky et al. (2011).

	This work	Tassinari “Office”	Difference (95% CI)	*p*	Tassinari “School”	Difference (95% CI)	*p*	Orlansky	Difference (95% CI)	*p*
*n*	115	53			13			181		
VT	0.932	0.96	0.028	n.s.	0.85	-0.082	n.s.	0.83	-0.102	<0.0001
			(-0.013 to 0.059)			(-0.356 to 0.023)			(-0.236 to -0.019)	
AHT	0.892	0.92	0.028	n.s.	0.89	-0.002	n.s.	0.78	-0.112	<0.005
			(-0.032 to 0.084)			(-0.216 to 0.085)			(-0.291 to -0.005)	
Ratio	0.649	0.76	0.111	n.s.	0.59	-0.059	n.s.	0.49	-0.159	0.05
			(-0.062 to 0.263)			(-0.582 to 0.229)			(-0.462 to 0.085)	
Errors	0.692	0.79	0.098	n.s.	0.44	-0.252	n.s.	0.39	-0.306	<0.0005
			(-0.058 to 0.234)			(-0.7 to 0.112)			(-0.585 to -0.042)	


**VT, vertical time; AHT, adjusted horizontal time*.*

Independent of the correlation used the results of the present study show significantly higher repeatability compared with other studies. Only with the Tassinari “school” group are there no significant differences, and the higher number of subjects involved in the present study confirms the previous result.

### Agreement

An efficient way to verify the agreement is to use the Bland and Altman limits of agreement graphical analysis and its associated statistics (Bland and Altman, 1986). In **Table 5**, we have listed the limits of agreement with the 95% upper and lower limits, with the 95% confidence limits.

**Table 5:** Limits of Agreement for the DEM subtest stratified by grades.

Grade	Subtest	Lower limit (95% CI)	Mean difference (95% CI)	Upper limit (95% CI)
2	VT	-9.28 (-11.92 to -6.64)	-2.04 (-3.57 to -0.52)	5.2 (2.56 to 7.84)
2	AHT	-30.05 (-38.24 to -21.86)	-7.59 (-12.32 to -2.86)	14.87 (6.68 to 23.07)
2	Ratio	-0.52 (-0.67 to -0.36)	-0.1 (-0.19 to -0.01)	0.32 (0.17 to 0.48)
2	Errors	-14.96 (-21.36 to -8.55)	2.6 (-1.1 to 6.3)	20.16 (13.75 to 26.56)
3	VT	-9.06 (-11.42 to -6.7)	-2.04 (-3.4 to -0.68)	4.98 (2.62 to 7.34)
3	AHT	-18.75 (-22.98 to -14.52)	-6.17 (-8.61 to -3.73)	6.42 (2.19 to 10.65)
3	Ratio	-0.34 (-0.43 to -0.25)	-0.08 (-0.13 to -0.03)	0.19 (0.1 to 0.27)
3	Errors	-14.55 (-18.83 to -10.27)	-1.83 (-4.3 to 0.64)	10.9 (6.62 to 15.17)
4	VT	-8.2 (-10.69 to -5.72)	-0.39 (-1.83 to 1.04)	7.42 (4.93 to 9.91)
4	AHT	-14.61 (-18.25 to -10.96)	-3.17 (-5.27 to -1.07)	8.27 (4.62 to 11.91)
4	Ratio	-0.34 (-0.42 to -0.25)	-0.06 (-0.11 to -0.01)	0.21 (0.12 to 0.3)
4	Errors	-7.84 (-10.37 to -5.3)	0.12 (-1.34 to 1.59)	8.09 (5.55 to 10.62)
5	VT	-10.19 (-12.92 to -7.46)	-2.06 (-3.64 to -0.48)	6.07 (3.34 to 8.81)
5	AHT	-12.48 (-15.07 to -9.9)	-4.79 (-6.29 to -3.3)	2.89 (0.31 to 5.48)
5	Ratio	-0.25 (-0.3 to -0.19)	-0.07 (-0.11 to -0.04)	0.1 (0.04 to 0.16)
5	Errors	-10.59 (-13.48 to -7.7)	-2 (-3.67 to -0.33)	6.59 (3.7 to 9.48)


**In each column the lower, mean and upper limits of agreement are reported, together with their specific ±95% CI intervals. (The units of these raw data are seconds for vertical time and adjusted horizontal time and number for errors). VT, vertical time; AHT, adjusted horizontal time.**

**FIGURE 1:**
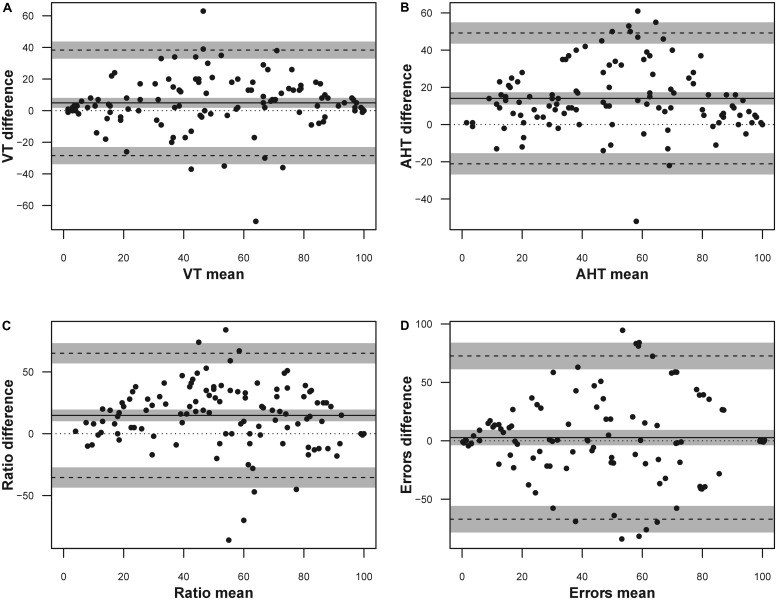
Bland Altman plot for the DEM subtests expressed as percentile scores. The solid line represents the mean difference, the dashed lines represent the upper and lower boundaries of the 95% limits of agreement, the gray areas represent the 95% confidence intervals of the limits of agreement (LoA). **(A)** Vertical time; **(B)** adjusted horizontal time; **(C)** ratio; **(D)** errors. Only the errors data were jittered (*x* ± 1; *y* ± 1) to visualize point density.

Because the limits of agreement calculation could also be performed with transformed data (Giavarina, 2015), we carried out these analyses with percentiles. The results are listed in **Table 6** and shown in **Figure 1**.

**Table 6:** Limits of Agreement for the DEM subtest expressed in percentile score.

	Lower limit (95% CI)	Mean difference (95% CI)	Upper limit (95% CI)
VT	-28.45 (-33.9 to -23)	4.93 (1.78 to 8.08)	38.31 (32.86 to 43.76)
AHT	-21.11 (-26.85 to -15.37)	14.05 (10.74 to 17.37)	49.22 (43.48 to 54.96)
Ratio	-35.44 (-43.64 to -27.23)	14.81 (10.07 to 19.54)	65.05 (56.85 to 73.26)
Errors	-67.12 (-78.53 to -55.72)	2.75 (-3.84 to 9.33)	72.62 (61.21 to 84.02)


**In each column the lower, mean and upper limits of agreement are reported, together with their specific ±95% CI intervals. (The values are listed as percentiles). VT, vertical time; AHT, adjusted horizontal time.**

**FIGURE 2:**
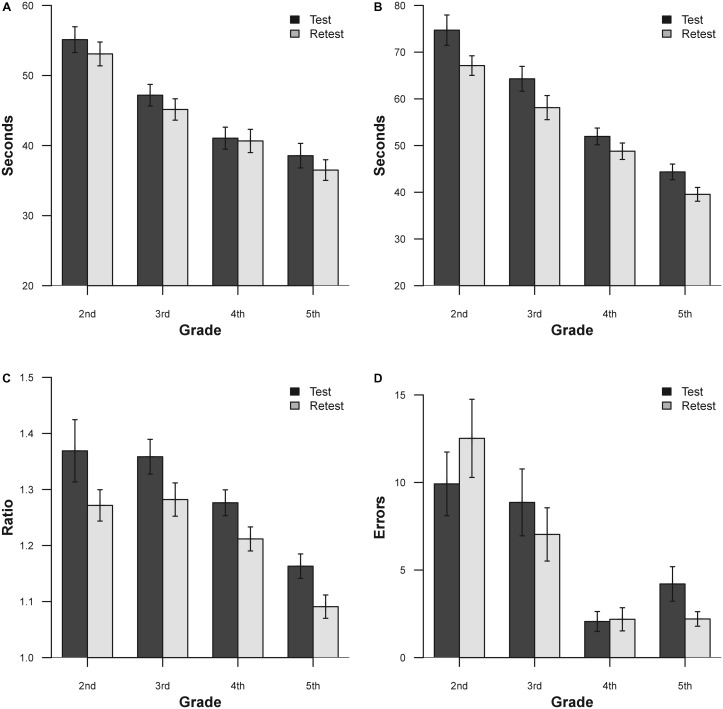
Mean results for the two separate sessions for DEM subtest and grade. **(A)** Vertical time; **(B)** adjusted horizontal time; **(C)** ratio; **(D)** errors. Bars represent ±1.

Another way to view the bias between sessions is to observe the mean and SD for vertical time, adjusted horizontal time, and ratio score for each age group are listed in **Table 7** and presented in **Figure 2**.

**Table 7:** Descriptive statistics for test and re-test data on the four subtests of DEM.

Subtest	Grade	Test mean	*SD*	Retest mean	*SD*
VT	2	55.12	9.19	53.08	8.5
	3	47.19	8.32	45.15	8.21
	4	41.05	8.9	40.66	9.4
	5	38.56	9.44	36.5	7.93
AHT	2	74.71	16.23	67.12	10.48
	3	64.29	14.35	58.12	13.92
	4	51.96	10.12	48.79	9.95
	5	44.35	9.05	39.56	7.95
Ratio	2	1.37	0.28	1.27	0.14
	3	1.36	0.17	1.28	0.16
	4	1.28	0.13	1.21	0.12
	5	1.16	0.12	1.09	0.11
Errors	2	9.92	9.09	12.52	11.15
	3	8.86	10.28	7.03	8.19
	4	2.06	3.21	2.19	3.74
	5	4.21	5.31	2.21	2.24


**VT, vertical time; AHT, adjusted horizontal time.**

Apart from the errors in grades 2 and 4, there is an evident improvement in performance on the second administration of the test. In order to verify this improvement, a series of ANOVA for each DEM subtest was performed. ANOVA was performed with one factor within (Time, with two levels), and one factor between (Grade, with four levels). The results for vertical time show significance for the factor Grade [*F*_(3,111)_ = 19.24, *p* < 0.0001, $\eta_{p}^{2}$ = 0.34] and for the factor Time [*F*_(1,111)_ = 19.33, *p* < 0.0001, $\eta_{p}^{2}$ = 0.16]. The adjusted horizontal time results show significance for the factor Grade [*F*_(3,111)_ = 35.04, *p* < 0.0001, $\eta_{p}^{2}$ = 0.48] and for the factor Time [*F*_(1,111)_ = 61.8, *p* < 0.0001, $\eta_{p}^{2}$ = 0.37]. The results for ratio show significance for the factor Grade [*F*_(3,111)_ = 12.08, *p* < 0.0001, $\eta_{p}^{2}$ = 0.25] and for the factor Time [*F*_(1,111)_ = 31.75, *p* < 0.0001, $\eta_{p}^{2}$ = 0.22]. The results for errors show significance for the factor Grade [*F*_(3,111)_ = 11.76, *p* < 0.0001, $\eta_{p}^{2}$ = 0.24] and for the interaction Time × Grade [*F*_(3,111)_ = 3.62, *p* < 0.05, $\eta_{p}^{2}$ = 0.08]. Across all grades, the subjects showed improvements with retest for vertical time, adjusted horizontal time and ratio scores (except for 2nd and 4th grade error; see **Table 7** for details).

In order to show the mean improvement of performance on retest in a different way, it is possible to view these results in terms of learning effect according to raw data and percentile improvement. The learning effect during sessions performed for each DEM subtest and grade (from 2nd to 5th) shows an improvement, respectively, of: 3.7, 4.32, 0.95, and 5.34% for vertical time, 10.16; 9.6; 6.10; and 10.8% for adjusted horizontal time, 7.3; 5.88; 5.47; and 6.03% for ratio and -26.21; 20.65; 6.31; and 47.51% for Errors. In percentile terms, the same results (unstratified) correspond to 4.93% for vertical time, 14.05% for adjusted horizontal time, 14.81% for ratio and 2.75% for errors. Lastly we report the standard error of measurement for all tests: 40.95 s for vertical time, 50.01 s for adjusted horizontal time, 0.301 for ratio and 14.44 for errors.

The pass-fail criteria for both administrations were only applied using the specific Italian norms for the 16th percentile criterion (Facchin et al., 2012).

The results listed in **Table 8** show a high or medium to high level of agreement for binary classification for vertical time, adjusted horizontal time, ratio and error. The same data of agreement reported in percentage show a range between 88 and 97% for vertical time, between 84 and 93% for adjusted horizontal time, between 75 and 97% for ratio and between 72 and 79% for errors. This level of agreement of binary classification appears to be equal or higher when compared with other studies, probably because it uses the last criterion of the 16th percentile (Tassinari and DeLand, 2005; Orlansky et al., 2011). Based on these data, we performed the Cohen *K* and AC_1_ as a measure of agreement. The results of Cohen *K* are listed in **Table 9**. These results on Cohen *K* are moderate to high for vertical time and low to moderate for adjusted horizontal time, ratio and errors. These values are lower than others that have been previously reported (Tassinari and DeLand, 2005), but the different criterion used (16th vs. 30th percentile) may explain the differences. The AC_1_ coefficients of agreement (±95% CI) were 0.89 (0.81 – 0.96) for vertical time, 0.84 (0.75 – 0.92) for adjusted horizontal time, 0.79 (0.69 – 0.90) for ratio and 0.59 (0.44 – 0.74) for error.

**Table 8:** Agreement between sessions separated for subtest (top) and grades (left), based on classification of the DEM findings (16th percentile).

			Retest							
			
			VT		AHT		Ratio		Error	
						
Test	Grade		P	F	P	F	P	F	P	F
	2nd	P	20	0	21	0	20	0	13	5
		F	2	3	4	0	5	0	2	5
	3rd	P	25	0	22	0	22	0	17	2
		F	1	3	4	3	4	3	4	6
	4th	P	24	3	26	2	22	2	23	2
		F	1	4	3	1	6	2	6	1
	5th	P	22	2	25	0	26	0	18	1
		F	0	5	2	2	1	2	7	3


**P, pass; F, fail. VT, vertical time; AHT, adjusted horizontal time.**

**Table 9:** Cohen’s *K* comparison between this study and those of Tassinari and DeLand (2005).

	This work	Tassinari “Office”	Difference (95% CI)
*n*	115	53	
VT	0.72 (0.55 to 0.89)	0.56 (0.34 to 0.79)	-0.16 (-0.438 to 0.126)
AHT	0.38 (0.14 to 0.62)	0.77 (0.56 to 0.98)	0.39 (0.071 to 0.709)
Ratio	0.37 (0.15 to 0.58)	0.73 (0.48 to 0.98)	0.36 (0.034 to 0.693)
Errors	0.36 (0.16 to 0.53)	0.77 (0.28 to 0.74)	0.41 (-0.109 to 0.612)


**VT, vertical time; AHT, adjusted horizontal time*.*

## Discussion

The purpose of this study was to re-evaluate the reliability of the DEM test with a test–retest method applying the original test (as used in practice) twice, scored in seconds and percentile and evaluating in depth the improving of performance between sessions. It is worth noting that the replication of experiments and confirmation of the results play an important role in science (Open Science Collaboration, 2015; Gelman and Geurts, 2017). One of the purposes of the present study was to perform a replication study in the context of another population and language and also using different norms.

Taking into account the strict definition of reliability as the correlation between test and retest, we have obtained results that are consistent with some studies that have reported high values (Rouse et al., 2004; Tassinari and DeLand, 2005), and our results are significantly higher than others (Orlansky et al., 2011), probably for the use the same test cards and which are not different parallel versions. In fact, we have reconfirmed the conclusions of previous studies for the good to excellent reliability for vertical and adjusted horizontal time but a medium to high reliability for ratio and error scores. On the other hand, it seems that the parallel and test–retest reliabilities are slightly different, with higher results for the latter which, in practice, is the most important because the original parallel forms are not practical available for this test.

The results of agreement analyses show that there is a significant and distinct trend in the amelioration of performance in the second repetition. This lack of agreement and the presence of a learning effect is the main problem with reliability of the DEM test.

Based on the previous well-known phenomenon of the learning effect, the main focus of our study was to calculate these results as percentile scores, besides confirming the phenomena using a different population and language. In fact, for monitoring the performance of a child over time or the use of the DEM test to assess the effectiveness of a therapy, there is a requirement to take into account the reliability of the test and its learning effect. The changes found in a second repetition of the test need to be greater than the repeatability itself. Our results, as expressed in seconds, show that, in order to be sure that the changes in the second administration can be attributed to therapy rather than test–retest variability, the results need to be higher than: about 9 s for the 2nd and 3rd grade, about 8 s for 4th and 10 s for 5th grade for vertical time; 30 s for 2nd grade, about 19 s for 3rd, about 15 s for 4th grade and 12 s for 5th grade for horizontal time; 0.5 for 2nd grade, 0.3 for 3rd and 4th grade and 0.25 for 5th grade for ratio; 15, 15, 8, and 11 errors, respectively, for 2nd, 3rd, 4th, and 5th grade for errors. These results are objectively weak but lower than the previously found which suggested 20 s for vertical time; 60 s for adjusted horizontal time, 0.6 for ratio, and 23 for errors, respectively (Orlansky et al., 2011). Moreover, we calculated not only the 95% limits of agreement, but also the 95% confidence interval to have a statistical confidence for this measure. Also with considering the confidence intervals, the difference between the results obtained by Orlansky et al. (2011) did not change (see **Table 6**).

Using percentile scoring, a score useful in practice, a change lower than 39 percentile points for vertical time, 49 for adjusted horizontal time, 65 for ratio and 72 for error as indicated could be interpreted, with care, to confirm amelioration. These values reflect the previous scores (limits of agreement) translated as percentiles and are useful for direct and easy clinical application. Confidence intervals on limits of agreement are calculated also for percentile scoring and reported in **Table 7**.

The lack of agreement and a remarkable learning effect was reflected in the generally moderate agreement of binary classification between sessions, with some changes in classification. The Kappa indexes of agreement were moderate to low and smaller than previously found. The AC_1_ index gave better results and part of the low scores in kappa could arise from the limitation of this index when data are highly asymmetrical. Nevertheless all these values have to be taken into account for clinical use. The improvement over sessions is the main problem with DEM test reliability, but knowing and quantifying it could permit the correct decisions to be taken when different sessions need to be compared.

A possible source of the aforementioned learning effect could be the lack of a true pre-test on DEM, especially in the first session (Facchin et al., 2014). Indeed, the manual reports that, in cases of doubt, the test needs to be performed twice, although the normative data were only collected for the first application and the improvement of time was not considered in the norms (Richman, 2009).

## Conclusion

Developmental eye movement test reliability has some limitations due to the lack of agreement between sessions, but our results show that this problem is lower than previously found. We have confirmed that the results should be evaluated carefully when the DEM test is used in monitoring the effectiveness of treatment with new values in seconds and percentiles. With awareness of this limitation, the DEM test can be used in clinics in performing ocular movement assessment over time from the professionals interested in vision assessment.

## Ethics Statement

We obtain the authorization from the “Istituto scolastico comprensivo “V.Muzio”, Via S.Pietro ai Campi 1, 24126 Bergamo, Italy” School Council to perform the screening and the research. They act as a control council over the all activities performed in the school. The authorization has a reference number 23/2010 and was obtained on April 5, 2010.

Regarding the informed consent, we asked at parents (or tutor) of the child to compile and sign the written informed consent.

Only children with written informed consent from their parents participated in the study.

Children without written informed consent were not admitted.

## Author Contributions

The authors contributed differently for the several aspects of this study. AF and SM conceptualization, methodology, writing – original draft, and writing – review and editing. AF data curation and formal analysis. SM investigation and supervision. All authors approved the final version of the work.

## Conflict of Interest Statement

The authors declare that the research was conducted in the absence of any commercial or financial relationships that could be construed as a potential conflict of interest.

## References

[B1] Altman D. G. Bland J. M. (1983). Measurement in medicine: the analysis of method comparison studies. *Stat. Methods Med. Res.* 32 307–317. 10.2307/2987937

[B2] Anastasi A. Urbina S. (1997). *Psychological Testing*, 7th Edn Prentice Hall, NJ: Upper Saddle River.

[B3] Associazione Italiana Dislessia (2007). *Learning Disabilities. Recommendations for Clinical Practice Defined with Consensus Conference.* Bologna: Associazione Italiana Dislessia.

[B4] Ayton L. N. Abel L. A. Fricke T. R. McBrien N. A. (2009). Developmental eye movement test: what is it really measuring? *Optom. Vis. Sci.* 86 722–730. 10.1097/OPX.0b013e3181a6a4b3 19417709

[B5] Baptista A. M. de Sousa R. A. de Morais Guerra Casal C. C. Marques R. J. da Silva C. M. (2011). Norms for the developmental eye movement test for portuguese children. *Optom. Vis. Sci.* 88 864–871. 10.1097/OPX.0b013e3182195dae 21499164

[B6] Bland J. M. Altman D. (1986). Statistical methods for assessing agreement between two methods of clinical measurement. *Lancet* 327 307–310. 10.1016/S0140-6736(86)90837-8 2868172

[B7] Costa Santos C. Bernardes J. Ayres-de-Campos D. (2011a). Observer reliability and agreement: differences, difficulties, and controversies. *J. Clin. Epidemiol.* 64 701–702. 10.1016/j.jclinepi.2010.12.00221411278

[B8] Costa Santos C. Bernardes J. Ayres-de-Campos D. Costa A. Costa C. (2011b). The limits of agreement and the intraclass correlation coefficient may be inconsistent in the interpretation of agreement. *J. Clin. Epidemiol.* 64 264–269. 10.1016/j.jclinepi.2009.11.010 20189765

[B9] Coulter R. A. Shallo-Hoffmann J. (2000). The presumed influence of attention on accuracy in the developmental eye movement (DEM) test. *Optom. Vis. Sci.* 77 428–432. 10.1097/00006324-200008000-00010 10966069

[B10] Diedenhofen B. Musch J. (2015). cocor: a comprehensive solution for the statistical comparison of correlations. *PLoS One* 10:e0121945. 10.1371/journal.pone.0121945 25835001 PMC4383486

[B11] Dormer A. Zou G. (2002). Interval estimation for a difference between intraclass kappa statistics. *Biometrics* 58 209–215. 10.1111/j.0006-341X.2002.00209.x 11890316

[B12] Facchin A. Maffioletti S. Carnevali T. (2011). Validity reassessment of developmental eye movement (DEM) test in the Italian population. *Optom. Vis. Dev.* 42 155–167.

[B13] Facchin A. Maffioletti S. Carnevali T. (2012). The developmental eye movement (DEM) test: normative data for Italian population. *Optom. Vis. Dev.* 43 162–179.

[B14] Facchin A. Ruffino M. Facoetti A. Maffioletti S. (2014). Modified direction of DEM test suggests differences in naming and eye movements. *Optom. Vis. Perform.* 2 103–111.

[B15] Fernandez-Velazquez F. J. Fernandez-Fidalgo M. J. (1995). Do DEM test scores change with respect to the language? Norms for Spanish-speaking population. *Optom. Vis. Sci.* 72 902–906. 10.1097/00006324-199512000-00009 8749338

[B16] Fleiss J. L. Cohen J. (1973). The equivalence of weighted kappa and the intraclass correlation coefficient as measures of reliability. *Educ. Psychol. Meas.* 33 613–619. 10.1177/001316447303300309

[B17] Fleiss J. L. Cohen J. Everitt B. S. (1969). Large sample standard errors of kappa and weighted kappa. *Psychol. Bull.* 72 323–327. 10.1037/h0028106 20816024

[B18] Garzia R. P. Richman J. E. Nicholson S. B. Gaines C. S. (1990). A new visual-verbal saccade test: the development eye movement test (DEM). *J. Am. Optom. Assoc.* 61 124–135. 2313029

[B19] Gelman A. Geurts H. M. (2017). The statistical crisis in science: how is it relevant to clinical neuropsychology? *Clin. Neuropsychol.* 31 1000–1014. 10.1080/13854046.2016.1277557 28075223

[B20] Giavarina D. (2015). Understanding Bland Altman analysis. *Biochem. Med.* 25 141–151. 10.11613/BM.2015.015 26110027 PMC4470095

[B21] Gwet K. L. (2008). Computing inter-rater reliability and its variance in the presence of high agreement. *Br. J. Math. Stat. Psychol.* 61 29–48. 10.1348/000711006X126600 18482474

[B22] Jimenez R. Gonzalez M. D. Perez M. A. Garcia J. A. (2003). Evolution of accommodative function and development of ocular movements in children. *Ophthalmic Physiol. Opt.* 23 97–107. 10.1046/j.1475-1313.2003.00093.x 12641697

[B23] Kline P. (2014). *The New Psychometrics: Science, Psychology and Measurement.* Milton Park: Taylor & Francis.

[B24] Kottner J. Streiner D. L. (2011). The difference between reliability and agreement. *J. Clin. Epidemiol.* 64 701–702. 10.1016/j.jclinepi.2010.12.001 21411278

[B25] McGraw K. O. Wong S. P. (1996). Forming inferences about some intraclass correlation coefficients. *Psychol. Methods* 1 30–46. 10.1037/1082-989X.1.1.30

[B26] Medland C. Walter H. Woodhouse M. J. (2010). Eye movements and poor reading: does the Developmental Eye Movement test measure cause or effect? *Ophthalmic Physiol. Opt.* 30 740–747. 10.1111/j.1475-1313.2010.00779.x 21205259

[B27] Okumura T. Wakamiya E. (2010). *Visual Skills in Children with Learning Difficulties.* Tokyo: Meijitosho Shuppan Corporation.

[B28] Open Science Collaboration (2015). Estimating the reproducibility of psychological science. *Science* 349:aac4716. 10.1126/science.aac4716 26315443

[B29] Orlansky G. Hopkins K. B. Mitchell G. L. Huang K. Frazier M. Heyman C. (2011). Reliability of the developmental eye movement test. *Optom. Vis. Sci.* 88 1507–1519. 10.1097/OPX.0b013e318230f03a 21964661

[B30] Palomo-Álvarez C. Puell M. C. (2009). Relationship between oculomotor scanning determined by the DEM test and a contextual reading test in schoolchildren with reading difficulties. *Graefes Arch. Clin. Exp. Ophthalmol.* 247 1243–1249. 10.1007/s00417-009-1076-8 19347678

[B31] Pang P. C. Lam C. S. Woo G. C. (2010). The Developmental Eye Movement (DEM) test and Cantonese-speaking children in Hong Kong SAR, China. *Clin. Exp. Optom.* 93 213–223. 10.1111/j.1444-0938.2010.00470.x 20465549

[B32] Powers M. Grisham D. Riles P. (2008). Saccadic tracking skills of poor readers in high school. *Optometry* 79 228–234. 10.1016/j.optm.2007.07.014 18436162

[B33] R Core Team (2017). *R: A Language and Environment for Statistical Computing.* Vienna: R Foundation for Statistical Computing.

[B34] Rainey B. B. Schroeder T. L. Goss D. A. Grosvenor T. P. (1998). Inter-examiner repeatability of heterophoria tests. *Optom. Vis. Sci.* 75 719–726. 10.1097/00006324-199810000-00016 9798211

[B35] Ramasundarahettige C. F. Donner A. Zou G. Y. (2009). Confidence interval construction for a difference between two dependent intraclass correlation coefficients. *Stat. Med.* 28 1041–1053. 10.1002/sim.3523 19142855

[B36] Richman J. E. (2009). *Developmental Eye Movement Test, Examiner’s Manual, version 2.0.* South Bend, IN: Bernell Corp.

[B37] Richman J. E. Garzia R. P. (1987). *Developmental Eye Movement Test, Examiners Booklet, version 1.* South Bend, IN: Bernell Corp.

[B38] Rouse M. W. Nestor E. M. Parot C. J. Deland P. N. (2004). A reevaluation of the Developmental Eye Movement (DEM) test’s repeatability. *Optom. Vis. Sci.* 81 934–938. 15592118

[B39] Santiago H. C. Perez M. A. (1992). “Test-retest reliability of the developmental eye movement test,” in *Proceedings of the AAO Annual Meeting*, Chicago, IL.

[B40] Scheiman M. Wick B. (2013). *Clinical Management of Binocular Vision: Heterophoric, Accommodative, and Eye Movement Disorders.* Philadelphia, PA: Lippincott Williams & Wilkins.

[B41] Serdjukova J. Ekimane L. Valeinis J. Skilters J. Krumina G. (2016). How strong and weak readers perform on the Developmental Eye Movement test (DEM): norms for Latvian school-aged children. *Read. Writ.* 30 233–252. 10.1007/s11145-016-9671-7

[B42] Tassinari J. T. DeLand P. (2005). Developmental Eye Movement Test: reliability and symptomatology. *Optometry* 76 387–399. 10.1016/j.optm.2005.05.006 16038866

[B43] Urbina S. (2004). *Essentials in Psychological Testing.* Hoboken, NJ: John Wiley and Sons.

[B44] Viera A. J. Garrett J. M. (2005). Understanding interobserver agreement: the kappa statistic. *Fam. Med.* 37 360–363. 15883903

[B45] Webber A. Wood J. Gole G. Brown B. (2011). DEM test, visagraph eye movement recordings, and reading ability in children. *Optom. Vis. Sci.* 88 295–302. 10.1097/OPX.0b013e31820846c0 21217407

[B46] Xie Y. Shi C. Tong M. Zhang M. Li T. Xu Y. (2016). Developmental Eye Movement (DEM) test norms for Mandarin Chinese-speaking Chinese children. *PLoS One* 11:e0148481. 10.1371/journal.pone.0148481 26881754 PMC4755595

[B47] Zou G. Y. (2007). Toward using confidence intervals to compare correlations. *Psychol. Methods* 12 399–413. 10.1037/1082-989X.12.4.399 18179351

